# Terahertz Kerr Effect of Liquids

**DOI:** 10.3390/s22239424

**Published:** 2022-12-02

**Authors:** Minghao Zhang, Wen Xiao, Cunlin Zhang, Liangliang Zhang

**Affiliations:** Key Laboratory of Terahertz Optoelectronics (MoE), Department of Physics, Capital Normal University, Beijing 100048, China

**Keywords:** terahertz wave, Kerr effect, liquid water, hydrogen bond, aqueous solution

## Abstract

In recent years, tremendous advancements have been made in various technologies such as far-infrared, low-frequency Raman, and two-dimensional (2D) Raman terahertz (THz) spectroscopies. A coherent method has emerged from numerous experimental and theoretical investigations of molecular dynamics in liquids by comparing linear and non-linear spectroscopic techniques. Intermolecular hydrogen bond vibration, molecular reorientation motion, and interaction between molecule/ionic solute and hydrogen bonds have been demonstrated to occur in the THz region, which are closely related to their physical/chemical properties and structural dynamics. However, precise probing of various modes of motion is difficult because of the complexity of the collective and cooperative motion of molecules and spectral overlap of related modes. With the development of THz science and technology, current state-of-the-art THz sources can generate pulsed electric fields with peak intensities of the order of microvolts per centimeter (MV/cm). Such strong fields enable the use of THz waves as the light source for non-linear polarization of the medium and in turn leads to the development of the emerging THz Kerr effect (TKE) technique. Many low-frequency molecular motions, such as the collective directional motion of molecules and cooperative motion under the constraint of weak intermolecular interactions, are resonantly excited by an intense THz electric field. Thus, the TKE technique provides an interesting prospect for investigating low-frequency dynamics of different media. In view of this, this paper first summarizes the research work on TKE spectroscopy by taking a solid material without low-frequency molecular motions as an example. Starting from the principle of TKE technology and its application in investigating the properties of solid matter, we have explored the low-frequency molecular dynamics of liquid water and aqueous solutions using TKE. Liquid water is a core of life and possesses many extraordinary physical and biochemical properties. The hydrogen bond network plays a crucial role in these properties and is the main reason for its various kinetic and thermodynamic properties, which differ from those of other liquids. However, the structure of the hydrogen bond network between water and solutes is not well known. Therefore, evaluating the hydrogen bond-related kinetic properties of liquid water is important.

## 1. Introduction

Terahertz (THz) pulses are characterized by low-photon energy, strong penetrability, and a frequency range covering the intermolecular vibrational and rotational energy levels of various organic/biological macromolecules. THz-related spectroscopic techniques play an important role in low-frequency molecular dynamics research [1,2,3,4,5,6], semiconductor and material property investigation [7,8,9,10,11,12,13,14,15], national defense and security [16,17,18], information technology [19,20,21,22,23,24], and medical diagnosis [25,26,27,28,29,30]. The Kerr effect describes the phenomenon of birefringence that occurs in a dielectric medium excited by an electric field [31]. After the development of the laser, the electric field of light was recognized to induce birefringence in the medium, which is called the optical Kerr effect (OKE) [32]. Hoffmann et al. later reported an intense single-cycle THz pulse-induced Kerr effect (TKE) in various liquids [33], which is a potential spectroscopic tool for exploring the underlying physical mechanisms. The successful acquisition of THz pulses with peak intensities above the order of MV/cm opens up a new field of non-linear THz spectroscopy. The TKE technique provides a solution for studying the instantaneous evolution of polarizability in media.

Water is the most important aspect of life. It can be combined with a variety of solutes to form solutions and more complex molecular motions. Aqueous solutions containing a variety of substances are closely related to various biochemical reactions in important fields, such as biomedicine and ecology. Low-frequency molecular motions associated with the hydrogen bond network in water and aqueous solutions play a crucial role in these biochemical reactions. Many thermodynamic and spectroscopic techniques present convincing insights and evidence of the bond energy, bond angle, bond length, and spatial structure of hydrogen bonds in liquid water. However, studying the molecular motion of liquid water in a higher temporal resolution has become more difficult [34,35]. The observation of the dynamic structure of hydrogen bonds with a high time resolution is essential for understanding the transient energy evolution of biochemical reactions in water or aqueous solutions [36,37,38,39,40,41,42,43,44,45,46]. Therefore, current research based on hydrogen bonds and their applications is still a promising frontier field. The motions associated with hydrogen bonds in liquid water are extremely sensitive to THz wave excitation. Therefore, the TKE technique is expected to achieve ultrafast time-resolved kinetic observations of hydrogen bond networks in liquid water. In addition, the THz electric field can resonate with a single-molecule rotational motion or low-frequency molecular motion, which in turn can result in enhanced molecular responses. Therefore, TKE spectroscopy enables clear fingerprints of low-frequency molecular motion modes in various substances, creating an interesting prospect for exploring low-frequency molecular dynamics in water and aqueous solutions.

The time-resolved TKE response, on a sub-picosecond scale, can identify the evolution of the dynamic properties of various molecular motion modes in liquid water. Some papers have outlined some of these contributions. However, no comprehensive paper has addressed the Kerr effect and its challenges and highlights potential applications. This paper introduces the latest progress and briefly explains the mechanism of TKE spectroscopy, which excites the third-order non-linear Kerr effect in various substances. This includes the observation of low-frequency molecular collective/cooperative motions associated with hydrogen bonds and kinetic theory of microscopic molecular dynamics in liquid water. A suitable theoretical model is proposed to explain the relationship between the molecular motion and polarizability anisotropy. The TKE technique is used to further explore the low-frequency molecular dynamics in aqueous solutions by adding ions and molecular solutes for analyzing the interaction between solutes and the hydrogen bond network of water molecules. This shows a new perspective for exploring the coupling and transfer of energy in the hydrogen bond network of liquid water and the interaction of molecules, ionic solutes, and hydrogen bond structures in aqueous solutions. It will provide new ideas to further explore the interaction between biological macromolecules and the hydrogen bond structure, influence of the hydrogen bond network on biochemical reactions, and biochemical reactions in aqueous environments within the THz frequency region.

## 2. TKE Technique Based on a Solid Medium

**Figure 1:**
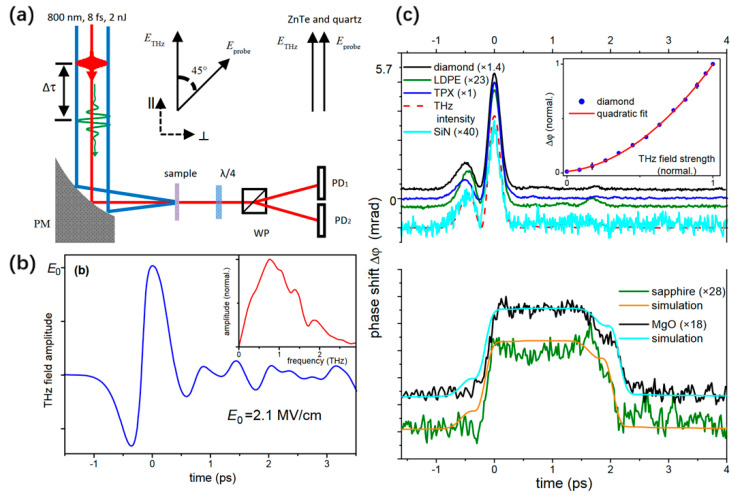
(**a**) Schematic of TKE experimental setup. (**b**) Time-domain waveform and corresponding spectrum of the THz wave. (**c**) TKE responses of diamond, low-density polyethylene (LDPE), polymethylpentene (TPX), silicon nitride (SiN), sapphire and magnesium oxide (MgO) windows [50].

Terahertz pulses with several MV/cm can be generated routinely through desktop sources. Such high fields open up the field of non-linear terahertz spectroscopy. Low-frequency modes of solids are driven into unexplored large-amplitude regions [47,48,49]. The dynamic evolution of the dielectric permittivity can be completely controlled by the THz electric field curve. However, obvious differences exist between the dielectric permittivity, owing to the optical propagation factors within the medium. Understanding and quantifying the distortion of the TKE response caused by the underlying linear optical parameters, such as dispersion and refractive index, is crucial for observing the evolution process of the third-order non-linear polarizability and further analyzing the low-frequency molecular dynamics in gas and liquid media. Sajadi et al. [50] measured the TKE response of common solid windows in the THz band and explored the influence of factors such as the phase mismatch in the medium. The experimental system and measured results are shown in Figure 1a,c, respectively. Δφ(t) is the phase difference between two orthogonal polarization components of the probe field, hitting the sample with a variable time-delay with respect to the pump [50]. Figure 1b shows the time-domain waveform and frequency spectrum of the excited THz wave using lithium niobate as a source. The effective frequency coverage was 0.1–2 THz. The measurements of several commonly used optical materials [50] provide basic data for the application of TKE spectroscopy.

**Figure 2:**
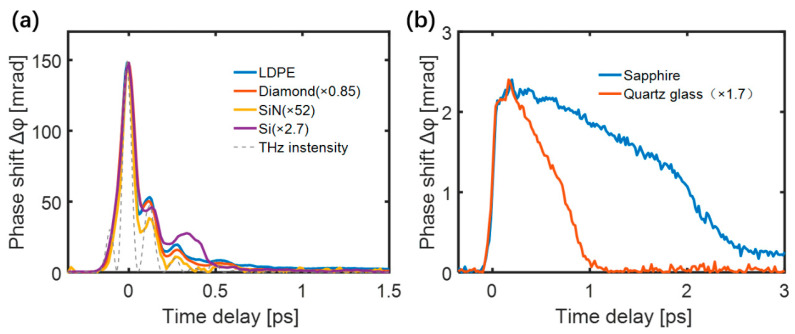
TKE responses of (**a**) LDPE, diamond, SiN, and silicon film. (**b**) sapphire and quartz.

Figure 2a shows the measured TKE responses of diamond (300 μm), low-density polyethylene (LDPE) (300 μm), Si_3_N_4_ (200 nm), and silicon thin film (10 μm) in which the THz intensity curve ($E_{THz}^{2}$) is also shown for comparison. The TKE response curves and traces of $E_{THz}^{2}$ have similar shapes. However, the TKE responses of the sapphire (0.5 mm) and quartz glass (0.5 mm) shown in Figure 2b have significantly different characteristics.

**Figure 3:**
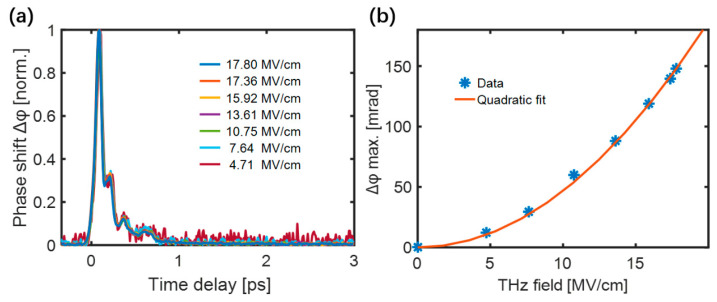
(**a**) The normalized responses of LDPE under different THz electric field strengths. (**b**) Dependence of the peak LDPE response on the THz electric field strength [51].

The maximum value of the transient birefringence was proportional to the square of the THz electric field for all samples. The normalized TKE responses of the low-density polyethylene (LDPE) excited by different THz electric field strengths are shown in Figure 3a. Figure 3b shows that the peak values of the responses were quadratically dependent on the THz electric field, proving the dominance of THz-induced third-order non-linear Kerr effect.

In these materials, the third-order polarizability generally originates from the polarization of the electron shell, where the low-frequency dynamics can be ignored. Therefore, the polarization response of matter can be considered to be instantaneous. The relationship between the transient birefringence Δn(z,t) induced by the THz electric field and collected phase difference Δφ(t) is as follows:

(1) $$\Delta \varphi \left(t\right)=\frac{2\pi }{\lambda }\int_{0}^{l}\Delta n\left(z,t\right)dz$$

where Δn(z,t) represents the change in the refractive index of the medium in the x and y directions, and can be defined along the two principal axes as

(2) $$\Delta n\left(z,t\right)=\Delta n_{x}\left(z,t\right)-\Delta n_{y}\left(z,t\right)=n_{2}I_{THz}=n_{2}c\varepsilon_{0}E_{THz}^{2}\left(z,t\right)$$

where *c* is the speed of light and $\varepsilon_{0}$ is the vacuum permittivity. The non-linear refractive index coefficient $n_{2}$ is derived from the intensity of the non-linear response, which is linearly related to the third-order non-linear polarizability tensor [52].

**Figure 4:**
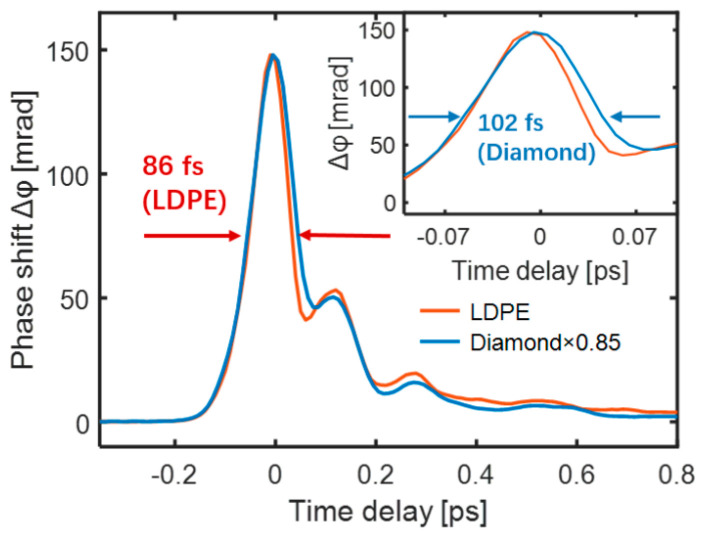
TKE responses of diamond (blue) and LDPE (red).

However, the measured TKE response could not completely follow the intensity curve $E_{THz}^{2}\left(x,t\right)$ of the input THz electric field. In particular, the quartz glass and sapphire shown in Figure 3b had wider response times. This is because Equation (2) describes the local relationship in an ideal section without considering the propagation of the THz and probe pulses inside the sample. In addition, the probe pulse had a pulse width. Therefore, signal acquisition was accompanied by sampling distortion resulting from the medium dispersion, refractive index, absorptivity, and thickness. LDPE is an ideal sample for THz wave-driven ultrafast non-linear effects owing to its large bandgap, small dispersion, and high THz transmittance. Diamond typically achieves ultrafast pulse switching on a femtosecond timescale [53]. The TKE responses of LDPE and diamond with the same thickness of 300 μm were measured and compared under the same experimental conditions. The two response curves exhibited similar temporal shapes, as shown in Figure 4. Compared with the ~102 fs gate time of diamond, LDPE exhibited a faster gate time of ~86 fs (FWHM) [51,54].

In addition to the response speed, the extinction ratio is another important factor in the evaluation of all-optical modulation devices. Compared with absorption modulation, polarization modulation is easier to achieve high extinction ratio [55,56]. Commonly used large-bandgap solid materials, such as LDPE (~8 eV) [57], diamond (~5.5 eV) [53], silicon nitride (>2 eV) [58], and sapphire (~6.7 eV) [59], are driven by strong THz electric fields (~MV/cm) without causing saturation distortion due to single-photon or multiphoton absorption in the material. Theoretically, these materials can achieve 90% extinction driven by a strong THz electric field. This section considered a solid material without low-frequency molecular dynamics as an example to analyze the measurement and factors that need to be considered in TKE spectroscopy and provided basic experimental data for the development of TKE applications.

## 3. TKE Responses of Liquid Water

Over the past few decades, an understanding of the structure, stability, and rearrangement dynamics of the hydrogen bond network in water has gradually developed [33,60,61,62,63,64,65,66]. A large number of experiments and theoretical studies have shown that strong collective and intermolecular cooperative motions of water are located in the far-infrared to THz region [67,68,69]. This provides a kinetic view of the hydrogen bonding structure in liquid water [33,69,70,71,72,73,74,75,76,77]. However, non-resonant OKE and Raman spectroscopy weakly perturbs the hydrogen bond network. Strong electron distortion and Rayleigh scattering background noise are collected together, which complicate the precise extraction of the hydrogen bond kinetics. The TKE provides a new way to investigate the ultrafast evolution of the Kerr effect. Hydrogen bonding vibrations and other molecular motions in aqueous solutions can also be characterized in the THz frequency region, which plays an important role in understanding the thermodynamic properties of liquids [78]. In 2015, Penkov et al. [79] analyzed the THz absorption spectra of liquid water and various aqueous solutions to characterize the collective dynamics of water molecules. In 2018, Ahmed et al. [80] used transient THz spectroscopy to study the interactions between hydrogen bond networks and excited dye molecules. Molecular motion in water has broad and fuzzy spectral lines in the THz range [6,81]. The analysis and observation of low-frequency molecular dynamics in liquid water or aqueous solutions using the TKE technique faces following three problems:

(1) Liquid water has a very strong absorption and THz waves have an extremely short penetration depth in water, resulting in a weak measurement. When the water film thickness is reduced to approximately 100 μm, the in–out ratio of THz photons to water can reach 9:1 (as shown in Table 1).

(2) The molecular dynamics, including the restricted translational, rotational, and diffusional motions of molecules, related to the hydrogen bond network in liquid water are more complex than those in benzene and carbon disulfide. Multiple molecular modes cover the entire THz frequency range. This complicates the assignment of these molecular motion patterns because of the weak signal response.

(3) Previous results [2,5,50,78], showed that common optical material windows exhibit strong TKE responses when driven by THz waves. This is sufficient to cover the TKE signal in liquid water, which makes acquiring the signal of water difficult.

**Figure 5:**
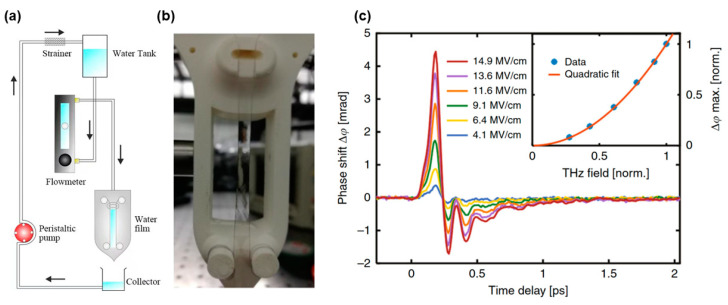
(**a**) Schematic of the water film device and (**b**) image of the water film. (**c**) TKE responses of water excited by THz pulses with different electric field strengths [1].

Therefore, gravity-driven free-flowing liquid devices have been exploited to generate thin, continuous, and stable water films [83]. Schematics and images are shown in Figure 5a,b, respectively. Figure 5c shows a set of bipolar signals with clear oscillatory properties of water excited by different THz electric field strengths. The responses were scaled with the square of the THz electric field, as shown in the inset, suggesting the dominance of Kerr effect. The broadband THz pulses excited two intermolecular motion modes: hydrogen bond bending and stretching vibrations. The positive signal was attributed to the hydrogen bond stretching vibration, and the negative signal was due to bending vibrations. Polarizability perturbation presents the competing contributions of the bending and stretching vibrations.

**Figure 6:**
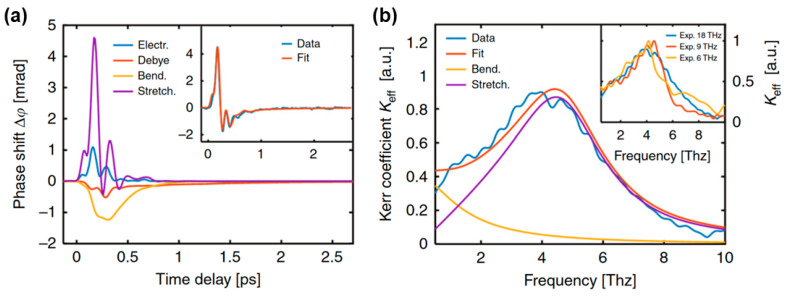
(**a**) Theoretical simulation results of the TKE response with electrons, hydrogen bond stretching and bending vibrations, and Debye relaxation. The inset shows the comparison between the sum of all contributions and measurement data. (**b**) Calculated frequency-domain Kerr coefficient of hydrogen bond bending (yellow line) and stretching (purple line) modes. The sum of two theoretical Kerr coefficients (red line) matches the experimental data with a cutoff frequency of 18 THz (blue line). The inset shows the measured Kerr coefficients extracted by cutoff frequencies of 18, 9 and 6 THz [1].

The Lorentz kinetic equation was used to simulate molecular motion. The equation is based on perturbations in the dielectric tensor caused by the intermolecular vibrational modes. The resulting polarizability anisotropy can be represented by the refractive index related to the dielectric susceptibility [1,52]:

(3) $$\begin{array}{c} \sum_{i=1,2}\Delta n_{i}=\frac{1}{2\varepsilon_{0}n_{0}}\left[\varepsilon_{y}-\frac{1}{2}\left(\varepsilon_{x}+\varepsilon_{z}\right)\right]=\frac{1}{2n_{0}}\left(\frac{\partial \alpha_{∥}}{\partial q_{2}}Q_{2}-\frac{\partial \alpha_{⊥}}{\partial q_{1}}Q_{1}\right) \end{array}$$

where $\varepsilon_{0}$ is the vacuum permittivity and $n_{0}$ is the refractive index of liquid water. Here, use $q_{1}$ and $q_{2}$ to represent the bending and stretching vibration amplitudes of a single hydrogen bond unit, respectively. $Q_{1}$ and $Q_{2}$ represent the anisotropic perturbations induced by hydrogen bond bending and stretching vibrations, respectively. $\alpha_{∥}$ and $\alpha_{⊥}$ denote the perturbation of the permittivity parallel and perpendicular to the direction of hydrogen bonding caused by intermolecular modes, respectively, which result in opposite birefringent contributions to the overall refractive index, that is, $\Delta n_{1}<0$, $\Delta n_{1}>0$. Furthermore, $Q_{i}$ satisfies the Lorentzian dynamics model, which describes the motion of a damped harmonic oscillator.

(4) $$\frac{\partial^{2}Q_{i}}{\partial t^{2}}+\gamma_{i}\frac{\partial Q_{i}}{\partial t}+\omega_{i}^{2}Q_{i}=a_{i}E^{2}\left(t\right),\;\left(i=1,2\right)$$

where $\gamma_{i}$ and $\omega_{i}$ represent the damping coefficients and frequencies of the hydrogen bond bending and stretching modes, respectively. In Raman spectroscopy, the typical values are: 3.45, 1.80, 5.10, and 5.70 THz [62,63]. Here, $a_{i}=\beta_{i}\left(\mu_{0}^{2}/3\sqrt{2}k_{B}Tm\right)$ represents the coupling factor between the square of the THz electric field and driving term in Equation (4). As shown in Figure 6a, the experimental data were decomposed and simulated into (i) positive responses of electron (blue line) and hydrogen bond stretching (purple lines) contributions and (ii) negative responses of Debye relaxation (red lines) and hydrogen bond bending (yellow lines) contributions [1]. This was verified by changing the THz frequency bandwidth using different low-pass filters. The simulation results agreed with the measured data. The positive response mainly originates from high-frequency molecular motion. This weak electron contribution can be attributed to the small hyperpolarization in water [2,84]. The frequency-domain Kerr coefficients *K_eff_* ($F\left(\Delta n\left(t\right)\right)/F\left(E^{2}\left(t\right)\right)/\lambda_{pr}$) were extracted from three excited THz pulses with cutoff frequencies of 18, 9, and 6 THz, as shown in Figure 6b. The Kerr coefficients mainly originate from the intermolecular modes. A prominent resonance peak appears around 4.5 THz, which corresponds to the hydrogen bond stretching vibrational mode. The hydrogen bond bending vibration is approximately a critically damped harmonic oscillator, resulting in no obvious peaks. The Kerr coefficient (blue line) can be approximated by the total contribution of the two intermolecular modes (red line).

## 4. Exploration of Molecular Dynamics by TKE Spectroscopy

**Figure 7:**
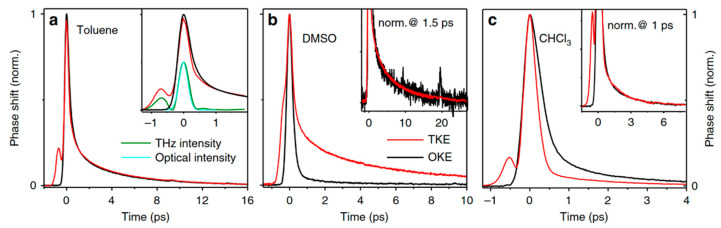
(**a**) TKE (red line) and OKE signal (black line) of (**a**) toluene, (**b**) DMSO, (**c**) CHCl_3_ [5].

Sajadi et al. [5] analyzed the collective orientation and relaxation dynamics of molecules on a sub-picosecond timescale by measuring the anisotropy of liquids, such as toluene, dimethyl sulfoxide (DMSO), and chloroform (CHCl_3_), driven by THz waves. The measurement results are shown in Figure 7. The TKE and OKE responses were compared in detail. The THz field-induced transient optical birefringence was more than one order of magnitude higher than optical excitation. This enhancement highlights the significance of THz field coupling to the permanent molecular dipole moments.

**Figure 8:**
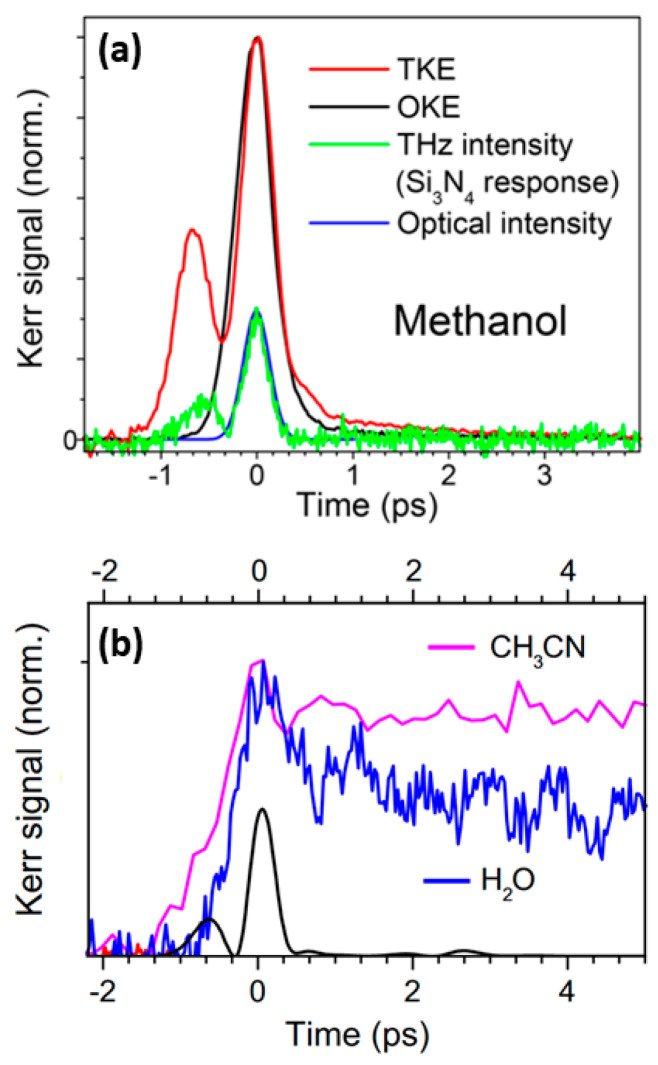
(**a**)TKE (red line) and OKE (black line) responses of methanol [3]. (**b**)TKE signals of water vapor (blue line) and acetonitrile (pink line). The black line represents instantaneous THz intensity [84].

Kampfrath et al. explored the molecular dynamics of alcoholic liquids [3]. They successfully measured the transient TKE response of methanol, as shown in Figure 8a. The rising peak was mainly attributed to the electronic contribution. Additionally, the relaxation of several picoseconds, resulting from the molecular response. This signal feature showed weak coupling between the THz electric field and permanent molecular dipole moment of the liquid.

**Figure 9:**
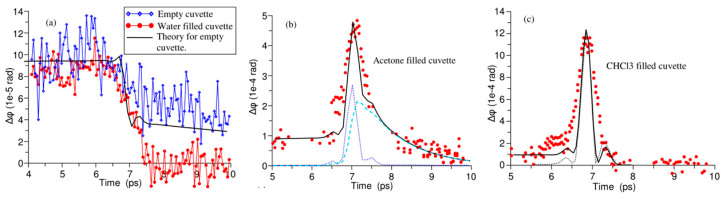
(**a**) TKE signals of water, (**b**) acetone, and (**c**) chloroform (red circles). The black lines represent the fitting curves [78]. Solid lines—theoretical calculation. Dotted and dashed lines in (**b**) show fast and slow Kerr responses, respectively. Dotted line in (**c**) traces THz pulse intensity.

Kampfrath et al. [84] further reported the TKE response of water vapor and stated the obvious long-time relaxation properties of the TKE signal of water vapor, as shown in Figure 8b. The response decreased to approximately half of the peak amplitude after approximately 5 ps. The relaxation process lasted for hundreds of picoseconds, and the TKE response of water vapor was mainly attributed to the molecular orientation. Bodrov et al. [78] compared the TKE responses of polar and non-polar liquids by measuring those of water, acetone, chloroform, carbon tetrachloride, and benzene. The measurement results are shown in Figure 9. Although they attempted to observe the molecular motion in liquid water, the experimental results were not ideal. Effective information on the molecular motion in liquid water was nearly obscured by the response of the sample cell.

**Figure 10:**
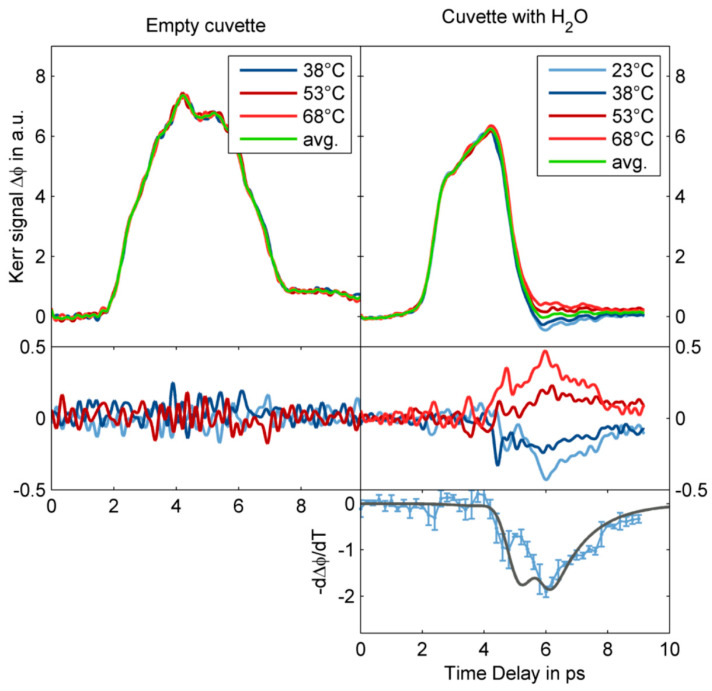
Temperature dependence of the TKE responses of an empty cuvette (left panels) and water inside the cuvette (right panels) [2].

Zalden et al. observed the transient orientation of the dipole moment of liquid water excited by a single cycle THz pulse centered at 0.25 THz generated from lithium niobate. Owing to the large TKE response of the sample cuvette, they extracted the response curve of water by changing temperature [2]. Figure 10 shows the TKE of water in a cuvette measured at various temperatures between 23 and 68 °C. Owing to the temperature independence of the electronic Kerr effect, the temperature-dependent signal originated from the molecular Kerr effect of water. The resulting (blue) curve agrees with the molecular contribution to the Kerr effect derived from modelling of the experimental data (black curve).

**Figure 11:**
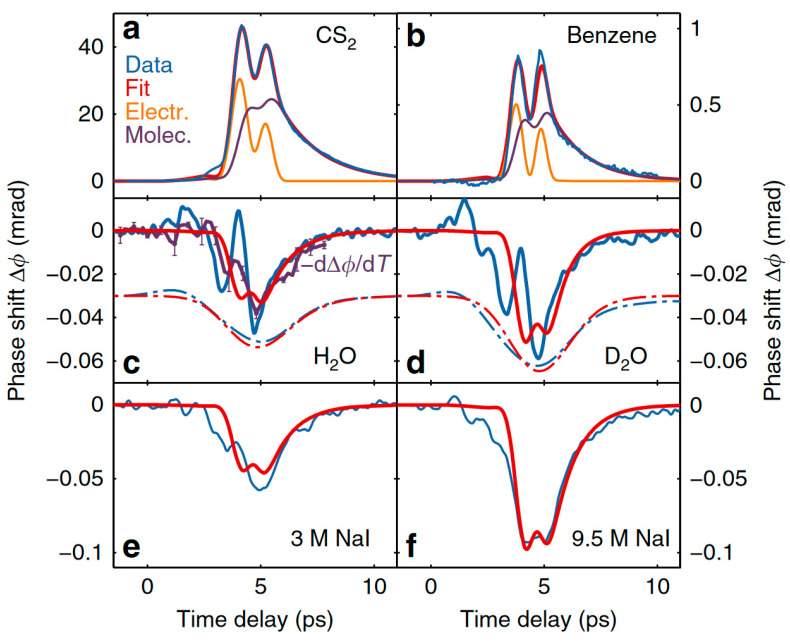
TKE responses of CS_2_, benzene, H_2_O, D_2_O, and NaI solutions [2].

They also measured the TKE responses of carbon disulfide (CS_2_), benzene, heavy water (D_2_O), and NaI solutions, as shown in Figure 11. Compared with the reference liquids, the response of water was extremely weak. They explained the mechanism of the collective directional movement of molecules based on the Langevin equation combined with the Kalmykov theory. The model was well supported by experimental data for CS_2_, benzene, methanol, and ethanol. However, the measurements of liquid water deviated from the theoretical speculation. Furthermore, the signal-to-noise ratio of the measured signal in water was too low to support an accurate kinetic analysis.

So far, the “direct” excitation of collective modes in Kerr measurements has been discussed. The technique can be also used to explore “indirect” excitations mediated, e.g., by anharmonicity. Elgabarty et al. demonstrated that the rotational energy in water molecules can be rapidly transferred to the confined translational motion of adjacent molecules under the excitation of a THz pulse (centered at 1 THz). This process can be explained by the strong anharmonicity of the intermolecular interactions [4]. However, they are limited by a narrowband low-frequency THz source, which complicates the complete observation of time-resolved intermolecular motions.

## 5. Low-Frequency Molecular Dynamics of Aqueous Solutions

Low-frequency molecular motion in aqueous solutions significantly influences biological activities, such as solvation, energy transfer, and proton transport. However, various anomalous properties arising from the underlying molecular motions in aqueous solutions are not well understood. Although numerous theoretical and experimental studies have been conducted on the physical properties of liquid water, the microscopic mechanism of the water hydrogen bond network under ionic perturbation has not been fully elucidated.

### 5.1. TKE Response of Salt Ion Solution

**Figure 12:**
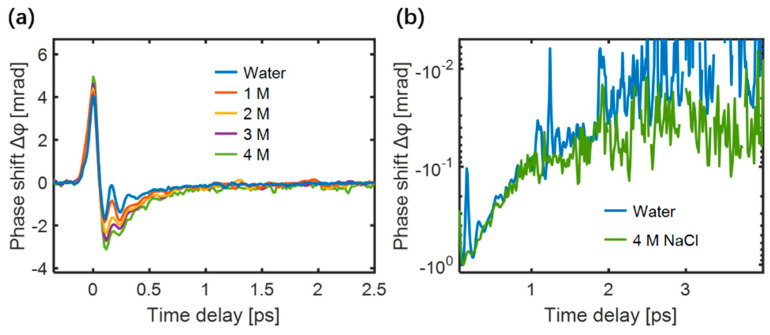
(**a**) TKE responses of NaCl solutions under different concentrations; (**b**) An enlarged logarithmic view of the negative polarity responses of water and 4 M NaCl solution [85].

The TKE responses of NaCl aqueous solutions were measured, as shown in Figure 12a. As the ion concentration increased, the characteristics of the TKE response exhibited three evident changes: (i) the response amplitude increased, (ii) the relaxation time increased, and (iii) the oscillation characteristics gradually smoothed. The normalized negative polarity responses of water and 4 M NaCl solution displayed a slight difference in the responses after 1 ps, as shown in Figure 12b, proving that the ions slow the recovery time of the water molecular motions.

**Figure 13:**
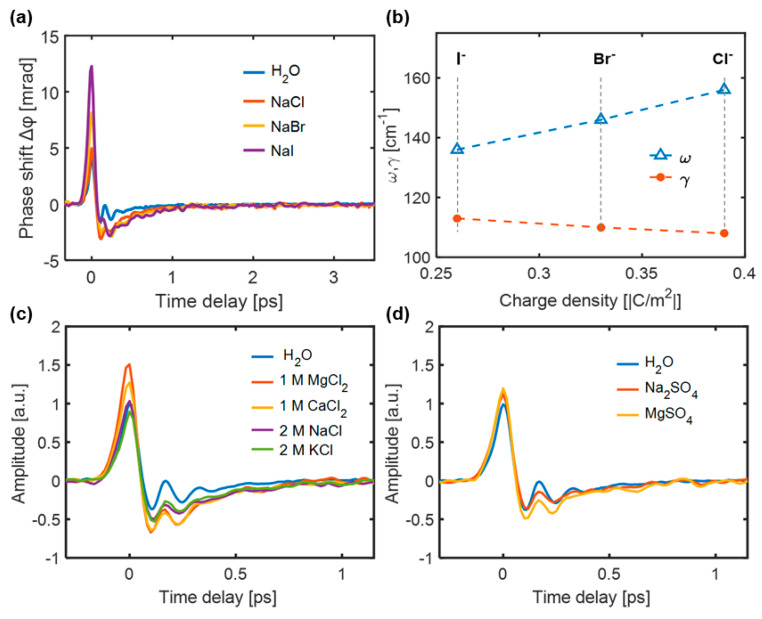
(**a**) TKE responses of water and 4 M NaCl, NaBr and NaI solutions. (**b**) Inherent frequency ω and damping coefficient γ [85]. TKE responses of (**c**) 1 M CaCl_2_ and MgCl_2_ aqueous solutions and 2 M NaCl and KCl and (**d**) 1 M Na_2_SO_4_ and MgSO_4_ aqueous solutions [87].

The TKE responses of the aqueous halide solutions were measured and are shown in Figure 13a. For the same concentration, the positive TKE response amplitude evidently increased in the order Cl^−^ < Br^−^ < I^−^, along with a decrease in the surface charge density of the anion. Notably, the NaI solution exhibits a higher anisotropic response because, for anions with low surface charge densities, such as I^−^, the formed OH···I^−^ hydrogen bond has a longer bond length and lower binding energy than the OH···Cl^−^ and OH···Br^−^ hydrogen bonds. The simulated inherent frequencies and damping coefficients of the OH···X^−^ (X^−^: Cl^−^, Br^−^, I^−^) hydrogen bond vibrational modes based on TKE spectroscopy are shown in Figure 13b. The obtained inherent frequencies and damping coefficients are consistent with the results obtained by non-resonant OKE spectroscopy [86]. Figure 13c shows the TKE responses of 1 M aqueous solutions of CaCl_2_ and MgCl_2_ and 2 M aqueous solutions of NaCl and KCl. Even though the molar concentrations of K^+^ and Na^+^ were twice those of Ca^2+^ and Mg^2+^, the increase in TKE response amplitude could still be arranged in the order of K^+^ < Na^+^ < Ca^2+^ < Mg^2+^. This implies that the effect of the cations on the TKE response was smaller than that of the anions. Figure 13b shows the TKE responses of 1 M Na_2_SO_4_ and MgSO_4_ aqueous solutions. The influence of Mg^2+^ was greater than that of Na^+^, even though the molar concentration of Na^+^ was twice that of Mg^2+^. This implies that the THz field couples more easily with the rotational and restricted translational motions of water molecules under the influence of strongly hydrated ions, such as in MgSO_4_ solution.

### 5.2. TKE Responses of Ethanol-Water Mixtures

**Figure 14:**
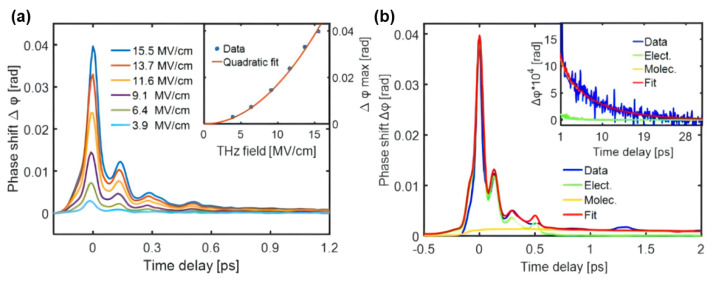
(**a**) TKE responses of ethanol excited by different THz electric field strengths. (**b**) TKE responses and simulated electronic and molecular responses of ethanol on sub-picosecond and picosecond timescales. The red line is the sum of the electronic and molecular contributions [91].

Ethanol is considered a representative molecule for investigating intermolecular interactions in biochemical processes. Exploring molecular motions in ethanol–water solutions with different mixing ratios is crucial for understanding the hydrogen bonding dynamics [88,89,90]. Figure 14a shows the TKE responses of ethanol excited by different THz electric field strengths. The signal has two characteristics: (i) a large response peak on a sub-picosecond timescale, and (ii) a slow decay process extending to tens of picoseconds. Usually, features (i) and (ii) mainly originate from the electronic and molecular responses, respectively. The simulated electronic response to ethanol [91] is indicated by the green line in Figure 14b. Moreover, the sum (red line) of simulated two kinds of responses is in good fit with the measured data (blue line). This means that the electron contribution plays a major role in the evolution process of sub-picosecond time scale, while the molecular contribution is dominant in the tens of picoseconds.

**Figure 15:**
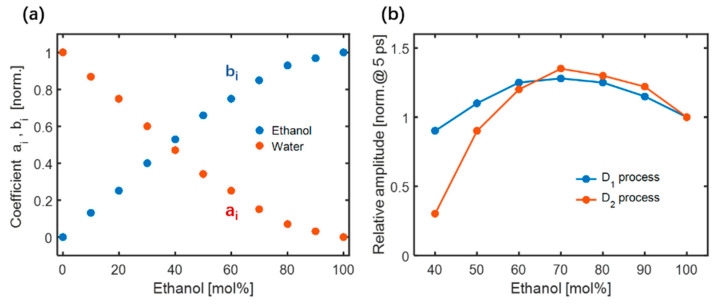
(**a**) The relationship between different molar concentrations and coefficients $a_{i}$ and $b_{i}$ within the sub-picosecond. (**b**) Relative amplitudes of the two Debye relaxation processes in mixtures of different molar concentrations [91].

The TKE responses of the ethanol–water mixtures at different concentrations were further explored. To analyze the relative effects of ethanol and water molecules in the mixture, the TKE responses of ethanol–water mixtures with different molar concentrations were fitted using Equation (5).

(5) $$\Delta \varphi t=a_{i}\Delta \varphi_{water}\left(t\right)+b_{i}\Delta \varphi_{ethanol}\left(t\right)$$

where $\Delta \varphi_{ethanol}\left(t\right)$ represents the TKE response of pure ethanol (*C*=100 mol%). Under the same conditions, it can achieve approximately 10 times the TKE response of pure water $\Delta \varphi_{water}\left(t\right)$. On a sub-picosecond timescale, Figure 15a shows the fitted values of $a_{i}$ and $b_{i}$. As the number of water molecules increased, their contribution to the total response increased, implying that the restricted translational motion of adjacent water molecules gradually contributed to the TKE responses of the mixture. A small number of ethanol molecules cannot affect the hydrogen bond network of water molecules in the mixture. However, the TKE response of the mixture is attributed to the water–water intermolecular hydrogen bond motion with the addition of ethanol molecules, indicating that the water intermolecular hydrogen bond network still contains anisotropy in the ethanol-rich region. These measurements of the ethanol–water mixture observed in the sub-picosecond time window were consistent with low-frequency Raman studies, showing that the intermolecular vibrational modes are independent of ethanol and water in the mixtures [92,93].

The coupling between the permanent molecule dipole moment and THz electric field can be assigned to two fast Debye relaxation processes. The D_1_ process involves the formation and breakage of hydrogen bonds with a relaxation time τ_1_ = 1–2 ps. The D_2_ process is related to the fluctuation of the terminal monomers of the hydrogen bond chain with τ_2_ = 7–12 ps. The molecular contributions to the TKE responses are described by a double exponential decay model, as shown in Figure 15b. The blue line represents the relative amplitude of the D_1_ process with respect to the molar concentration of the mixtures. A relatively enhanced D_1_ process was observed when a small number of water molecules were added to ethanol molecules. As the number of water molecules increased, the amplitude decreased. An inflection point appeared near a concentration of 70 mol%, which was attributed to the equilibrium of the molecular motions associated with the hydrogen bond breakage and formation of ethanol. The relative amplitude of the D_2_ process, depicted by the red line, increased as the molar concentration of ethanol decreased in the range of C =100–70 mol%. The addition of a small number of water molecules caused the long hydrogen bond chain of ethanol to break into several short chains, thereby increasing the number of terminal ethanol monomers in the chains and probability of the formation and breakage of ethanol intermolecular hydrogen bonds. As the number of water molecules increased (in the range of C = 70–30 mol%), the amplitude gradually decreased. The number of free ethanol monomers isolated by water molecules increased, resulting in a decrease in the proportion of terminal ethanol monomers in the chain. When the molar concentration of ethanol was less than 30 mol%, the amplitude of the D_2_ process quickly dropped to the noise level. This is primarily because ethanol molecular clusters are dissolved by water molecules, which makes it difficult to maintain the chain-like structure of ethanol. For more details, please refer to [91].

## 6. Conclusions and Outlook

TKE spectroscopy serves as a novel tool for directly observing the ultrafast time evolution of intermolecular hydrogen bond dynamics in water and various aqueous solutions. This paper reviews the related work on the development of the TKE technique from solid media to liquids. These studies realized the effective observation of hydrogen bond motion on the sub-picosecond timescale and proposed a theoretical model to explain the anisotropy caused by molecular motion. The TKE responses excited by intense broadband THz pulses provide a time-resolved perspective to explore the collective or cooperative motion of molecules in liquid water and aqueous solutions. This has promoted the development of microscopic mechanistic research on biological macromolecular dynamics. Research on the TKE technique mentioned in this paper lays the foundation for probing the birefringence induced by complex low-frequency molecular motions in matter.

Research on the low-frequency molecular motion of liquid water and aqueous solutions excited by THz waves is expected for future applications. For example, solvated water around biomacromolecules controls several important biological reactions. TKE spectroscopy can resonantly excite intermolecular hydrogen bonds, and the induced transient birefringence can reveal the intermolecular dynamics in water. Therefore, the TKE technique promises to directly observe kinetic features such as ligand–protein binding in aqueous protein solutions. The detection of THz waves in solids, gases, and plasmas has been extensively studied for decades. However, THz wave detection using liquids has not yet been achieved. In this study, the transient birefringence response of water driven by intense THz waves is reviewed, and the physical mechanism of the interaction between THz waves and liquid water is analyzed. This provides a new idea for the detection THz waves.

## Figures and Tables

**Table 1:** Transmission of THz photons by water films with different thicknesses [82].

Water Film Thickness d (μm)	Incident Number of THz Photons	The Exit Number of THz Photons
500	${6\;\times \;10}^{4}$	1
100	9	1
50	3	1

## Data Availability

Not applicable.

## References

[1] Zhao H., Tan Y., Zhang L., Zhang R., Shalaby M., Zhang C., Zhao Y., Zhang X.-C. (2020). Ultrafast hydrogen bond dynamics of liquid water revealed by terahertz-induced transient birefringence. Light Sci. Appl..

[2] Zalden P., Song L., Wu X., Huang H., Ahr F., Mücke O.D., Reichert J., Thorwart M., Mishra P.K., Welsch R. (2018). Molecular polarizability anisotropy of liquid water revealed by terahertz-induced transient orientation. Nat. Commun..

[3] Kampfrath T., Campen R.K., Wolf M., Sajadi M. (2018). The nature of the dielectric response of methanol revealed by the terahertz Kerr effect. J. Phys. Chem. Lett..

[4] Elgabarty H., Kampfrath T., Bonthuis D.J., Balos V., Kaliannan N.K., Loche P., Netz R.R., Wolf M., Kühne T.D., Sajadi M. (2020). Energy transfer within the hydrogen bonding network of water following resonant terahertz excitation. Sci. Adv..

[5] Sajadi M., Wolf M., Kampfrath T. (2017). Transient birefringence of liquids induced by terahertz electric-field torque on permanent molecular dipoles. Nat. Commun..

[6] Freysz E., Degert J. (2010). Terahertz Kerr effect. Nat. Photon..

[7] Bead M.C., Turner G.M., Schmuttenmaer C.A. (2002). Size -dependent photoconductivity in CdSe nanoparticles as measured by time-resolved terahertz spectroscopy. Nano Lett..

[8] Azad A.K., Zhang W. (2006). Terahertz dielectric properties of high-resistivity single-crystal ZnO. Appl. Phys. Lett..

[9] Tan Y., Zhao H., Zhang L., Zhang Y., Zhang C., Weber C., Acharya S., Cunningham B., Gruning M., Liu K. Possible phonon-induced electronic Bi-stability in VO2 for ultrafast memory at room temperature. Proceedings of the 2019 44th International Conference on Infrared, Millimeter, and Terahertz Waves (IRMMW-Thz).

[10] Zhang L., Chen Z., Zhang R., Tan Y., Wu T., Shalaby M., Xie R., Xu J. (2019). Direct observation of charge injection of graphene in the Graphene/WSe2 Heterostructure by optical-pump terahertz-probe spectroscopy. ACS Appl. Mater. Interfaces.

[11] Schmuttenmaer C.A. (2004). Exploring dynamics in the far-infrared with terahertz spectroscopy. Chem. Rev..

[12] Huber R., Tauser F., Brodschelm A., Bichler M., Abstreiter G., Leitenstorfer A. (2001). How many-particle interactions develop after ultrafast excitation of an electron-hole plasma. Nature.

[13] Weber C., Acharya S., Cunningham B., Gruning M., Zhang L., Zhao H., Tan Y., Zhang Y., Zhang C., Liu K. (2020). Role of the lattice in the light-induced insulator-to-metal transition in vanadium dioxide. Phys. Rev. Res..

[14] Chen Z., Chen X., Tao L., Chen K., Long M., Liu X., Yan K., Stantchev R.I., Pickwell-MacPherson E., Xu J.-B. (2018). Graphene controlled brewster angle device for ultra broadband terahertz modulation. Nat. Commun..

[15] Gopalan P., Sensale-Rodriguez B. (2020). 2D materials for terahertz modulation. Adv. Opt. Mater..

[16] Melinger J.S., Harsha S.S., Laman N., Grischkowsky D. (2010). Temperature dependent characterization of terahertz vibrations of explosives and related threat materials. Opt. Express.

[17] Ergün S., Sönmez S. (2015). Terahertz technology for military applications. J. Mil. Inf. Sci..

[18] Palka N., Szala M., Czerwinska E. (2016). Characterization of prospective explosive materials using terahertz time-domain spectroscopy. Appl. Opt..

[19] Jastrow C., Priebe S., Spitschan B., Hartmann J., Jacob M., Kuerner T., Schrader T., Kleine-Ostmann T. (2010). Wireless digital data transmission at 300 GHz. Electron. Lett..

[20] Song H.-J., Nagatsuma T. (2011). Present and future of terahertz communications. IEEE Trans. Terahertz Sci. Technol..

[21] Nagatsuma T., Ducournau G., Renaud C.C. (2016). Advances in terahertz communications accelerated by photonics. Nat. Photon..

[22] Sklar B. (2001). Digital Communications Fundamentals and Applications.

[23] Narayanan R.M., Chuang J. (2007). Covert communications using heterodyne correlation random noise signals. Electron. Lett..

[24] Kalinin V.I. (1996). Spectral modulation of wideband noise signals. Radioteknika Elektron..

[25] Woodward R.M., Wallace V.P., Pye R.J., Cole B.E., Arnone D.D., Linfield E.H., Pepper M. (2003). Terahertz pulse imaging of ex vivo basal cell carcinoma. J. Investig. Dermatol..

[26] Fitzgerald A.J., Berry E., Zinov’ev N.N., Homer-Vanniasinkam S., Miles R.E., Chamberlain J.M., Smith M.A. (2003). Catalogue of human tissue optical properties at terahertz frequencies. J. Biol. Phys..

[27] Scarfi M.R., Romano M., Di Pietro R., Zeni O., Doria A., Gallerano G.P., Giovenale E., Messina G., Lai A., Campurra G. (2003). THz exposure of whole blood for the study of biological effects on human lymphocytes. J. Biol. Phys..

[28] Oh S.J., Kim S.-H., Ji Y.B., Jeong K., Park Y., Yang J., Park D.W., Noh S.K., Kang S.-G., Huh Y.-M. (2014). Study of freshly excised brain tissues using terahertz imaging. Biomed. Opt. Express.

[29] Joseph C.S., Yaroslavsky A.N., Neel V.A., Goyette T.M., Giles R.H. (2011). Continuous wave terahertz transmission imaging of nonmelanoma skin cancers. Lasers Surg. Med..

[30] Cheon H., Yang H., Lee S.-H., Kim Y.A., Son J.-H. (2016). Terahertz molecular resonance of cancer DNA. Sci. Rep..

[31] Kampfrath T., Wolf M., Sajadi M. (2017). Anharmonic coupling between intermolecular motions of water revealed by terahertz Kerr effect. arXiv.

[32] Luzar A., Chandler D. (1996). Hydrogen-bond kinetics in liquid water. Nature.

[33] Perakis F., De Marco L., Shalit A., Tang F., Kann Z.R., Kuehne T.D., Torre R., Bonn M., Nagata Y. (2016). Vibrational spectroscopy and dynamics of water. Chem. Rev..

[34] Zhang C., Khaliullin R.Z., Bovi D., Guidoni L., Kuehne T.D. (2013). Vibrational signature of water molecules in asymmetric hydrogen bonding environments. J. Phys. Chem. Lett..

[35] Khaliullin R.Z., Kuehne T.D. (2013). Microscopic properties of liquid water from combined Ab initio molecular dynamics and energy decomposition studies. Phys. Chem. Chem. Phys..

[36] Paolantoni M., Sassi P., Morresi A., Santini S. (2007). Hydrogen bond dynamics and water structure in glucose-water solutions by depolarized rayleigh scattering and low-frequency Raman spectroscopy. J. Chem. Phys..

[37] Bizzarri A.R., Wang C., Chen W., Cannistraro S. (1995). Hydrogen bond analysis by MD simulation of copper plastocyanin at different hydration levels. Chem. Phys..

[38] Wu K., Qi C., Zhu Z., Wang C., Song B., Chang C. (2020). Terahertz wave accelerates DNA unwinding: A molecular dynamics simulation study. J. Phys. Chem. Lett..

[39] Hartnig C., Koper M.T.M. (2003). Solvent reorganization in electron and ion transfer reactions near a smooth electrified surface: A molecular dynamics study. J. Am. Chem. Soc..

[40] Ando K., Hynes J.T., Prigogine I., Rice S.A. (1999). Acid-base proton transfer and ion pair formation in solution. Advances in Chemical Physics.

[41] Halle B. (2004). Protein hydration dynamics in solution: A critical survey. Philos. Trans. R. Soc. B-Biol. Sci..

[42] Malardier-Jugroot C., Johnson M.E., Murarka R.K., Head-Gordon T. (2008). Aqueous peptides as experimental models for hydration water dynamics near protein surfaces. Phys. Chem. Chem. Phys..

[43] Mazur K., Heisler I.A., Meech S.R. (2010). Ultrafast dynamics and hydrogen-bond structure in aqueous solutions of model peptides. J. Phys. Chem. B.

[44] Born B., Weingaertner H., Bruendermann E., Havenith M. (2009). Solvation dynamics of model peptides probed by terahertz spectroscopy. Observation of the onset of collective network motions. J. Am. Chem. Soc..

[45] Modig K., Liepinsh E., Otting G., Halle B. (2004). Dynamics of protein and peptide hydration. J. Am. Chem. Soc..

[46] Qvist J., Persson E., Mattea C., Halle B. (2009). Time scales of water dynamics at biological interfaces: Peptides, proteins and cells. Faraday Discuss..

[47] Kampfrath T., Tanaka K., Nelson K.A. (2013). Resonant and nonresonant control over matter and light by intense terahertz transients. Nat. Photon..

[48] Tanaka K., Hirori H., Nagai M. (2011). THz nonlinear spectroscopy of solids. IEEE Trans. Terahertz Sci. Technol..

[49] Hebling J., Yeh K.L., Hoffmann M.C., Nelson K.A. (2008). High-power THz generation, THz nonlinear optics, and THz nonlinear spectroscopy. IEEE J. Sel. Top. Quantum Electr..

[50] Sajadi M., Wolf M., Kampfrath T. (2015). Terahertz-field-induced optical birefringence in common window and substrate materials. Opt. Express.

[51] Tan Y., Zhao H., Zhang R., Zhang C., Zhao Y., Zhang L. (2020). Ultrafast optical pulse polarization modulation based on the terahertz-induced Kerr effect in low-density polyethylene. Opt. Express.

[52] Boyd R.W. (2003). Nonlinear Optics.

[53] Shalaby M., Vicario C., Hauri C.P. (2017). Extreme nonlinear terahertz electro-optics in diamond for ultrafast pulse switching. APL Phonton..

[54] Smith D., Loewenstein E. (1975). Optical-constants of far infrared materials. 3: Plastics. Appl. Opt..

[55] Seren H.R., Keiser G.R., Cao L., Zhang J., Strikwerda A.C., Fan K., Metcalfe G.D., Wraback M., Zhang X., Averitt R.D. (2014). Optically modulated multiband terahertz perfect absorber. Adv. Opt. Mater..

[56] Chen H.-T., Padilla W.J., Zide J.M.O., Bank S.R., Gossard A.C., Taylor A.J., Averitt R.D. (2007). Ultrafast optical switching of terahertz metamaterials fabricated on ErAs/GaAs nanoisland superlattices. Opt. Lett..

[57] Fujihira M., Inokuchi H. (1972). Photoemission from polyethylene. Chem. Phys. Lett..

[58] Demichelis F., Crovini G., Giorgis F., Pirri C.F., Tresso E. (1996). Hydrogenated amorphous silicon-nitrogen alloys, a-SiNx:H-y: A wide band gap material for optoelectronic devices. J. Appl. Phys..

[59] Nohira H., Tsai W., Besling W., Young E., Petry J., Conard T., Vandervorst W., De Gendt S., Heyns M., Maes J. (2002). Characterization of ALCVD-Al2O3 and ZrO2 layer using X-Ray photoelectron spectroscopy. J. Non-Cryst. Solids.

[60] Vij J.K., Simpson D.R.J., Panarina O.E. (2004). Far infrared spectroscopy of water at different temperatures: GHz to THz dielectric spectroscopy of water. J. Mol. Liq..

[61] Torii H. (2011). Intermolecular electron density modulations in water and their effects on the far-infrared spectral profiles at 6 THz. J. Phys. Chem. B.

[62] Mizoguchi K., Hori Y., Tominaga Y. (1992). Study on dynamic structure in water and heavy-water by low-frequency Raman-spectroscopy. J. Chem. Phys..

[63] Fukasawa T., Sato T., Watanabe J., Hama Y., Kunz W., Buchner R. (2005). Relation between dielectric and low-frequency Raman spectra of hydrogen-bond liquids. Phys. Rev. Lett..

[64] Ronne C., Keiding S.R. (2002). Low frequency spectroscopy of liquid water using THz-time domain spectroscopy. J. Mol. Liq..

[65] Penkov N., Shvirst N., Yashin V., Fesenko E., Fesenko E. (2015). Terahertz spectroscopy applied for investigation of water structure. J. Phys. Chem. B.

[66] Savolainen J., Ahmed S., Hamm P. (2013). Two-dimensional Raman-terahertz spectroscopy of water. Proc. Natl. Acad. Sci. USA.

[67] Soper A.K. (2000). The radial distribution functions of water and ice from 220 to 673 K and at pressures up to 400 MPa. Chem. Phys..

[68] Laage D. (2009). Reinterpretation of the liquid water quasi-elastic neutron scattering spectra based on a nondiffusive jump reorientation mechanism. J. Phys. Chem. B.

[69] Teixeira J., Bellissentfunel M., Chen S., Dianoux A. (1985). Experimental-determination of the nature of diffusive motions of water-molecules at low-temperatures. Phys. Rev. A.

[70] Greene B., Farrow R. (1983). The subpicosecond Kerr effect in Cs2. Chem. Phys. Lett..

[71] Waldeck D., Cross A., Mcdonald D., Fleming G. (1981). Picosecond pulse induced transient molecular birefringence and dichroism. J. Chem. Phys..

[72] Fecko C.J., Eaves J.D., Tokmakoff A. (2002). Isotropic and anisotropic Raman scattering from molecular liquids measured by spatially masked optical Kerr effect spectroscopy. J. Chem. Phys..

[73] Turton D.A., Wynne K. (2008). Structural relaxation in the hydrogen-bonding liquids N-methylacetamide and water studied by optical Kerr effect spectroscopy. J. Chem. Phys..

[74] Palese S., Schilling L., Miller R., Staver P., Lotshaw W. (1994). Femtosecond optical Kerr-effect studies of water. J. Phys. Chem..

[75] Skaf M.S., Sonoda M.T. (2005). Optical Kerr effect in supercooled water. Phys. Rev. Lett..

[76] Torre R., Bartolini P., Righini R. (2004). Structural relaxation in supercooled water by time-resolved spectroscopy. Nature.

[77] Hunt N.T., Kattner L., Shanks R.P., Wynne K. (2007). The dynamics of water-protein interaction studied by ultrafast optical Kerr-effect spectroscopy. J. Am. Chem. Soc..

[78] Bodrov S., Sergeev Y., Murzanev A., Stepanov A. (2017). Terahertz induced optical birefringence in polar and nonpolar liquids. J. Chem. Phys..

[79] Ahmed S., Pasti A., Fernández-Terán R.J., Ciardi G., Shalit A., Hamm P. (2018). Aqueous solvation from the water perspective. J. Chem. Phys..

[80] Ahmed S., Savolainen J., Hamm P. (2014). The effect of the Gouy phase in optical-pump-THz-probe spectroscopy. Opt. Express.

[81] Hoffmann M.C., Brandt N.C., Hwang H.Y., Yeh K.L., Nelson K.A. (2009). Terahertz Kerr effect. Appl. Phys. Lett..

[82] Rϕnne C., Thrane L., Åstrand P.O., Wallqvist A., Mikkelsen K.V., Keiding S.R. (1997). Investigation of the temperature dependence of dielectric relaxation in liquid water by THz reflection spectroscopy and molecular dynamics simulation. J. Chem. Phys..

[83] Wang T., Klarskov P., Jepsen P.U. (2014). Ultrabroadband THz time-domain spectroscopy of a free-flowing water film. IEEE Trans. Terahertz Sci. Technol..

[84] Kampfrath T., Wolf M., Sajadi M. (2018). The sign of the polarizability anisotropy of polar molecules is obtained from the terahertz Kerr effect. Chem. Phys. Lett..

[85] Zhao H., Tan Y., Zhang R., Zhao Y., Zhang C.L., Zhang L.L. (2021). Anion-water hydrogen bond vibration revealed by the terahertz Kerr effect. Opt. Lett..

[86] Collins K.D. (1995). Sticky ions in biological systems. Proc. Natl. Acad. Sci. USA.

[87] Zhao H., Tan Y., Wu T., Zhang R., Zhao Y.J., Zhang C.L., Zhang L.L. (2021). Strong anisotropy in aqueous salt solutions revealed by terahertz-induced Kerr effect. Opt. Commun..

[88] Zasetsky A.Y., Lileev A.S., Lyashchenko A.K. (2010). Molecular dynamic simulations of terahertz spectra for water-methanol mixtures. Mol. Phys..

[89] Li R., Agostino C.D., McGregor J., Mantle M.D., Zeitler J.A., Gladden L.F. (2014). Mesoscopic structuring and dynamics of alcohol/water solutions probed by terahertz time-domain spectroscopy and pulsed field gradient nuclear magnetic resonance. J. Phys. Chem. B.

[90] Sani E., Dell’Oro A. (2016). Spectral optical constants of ethanol and isopropanol from ultraviolet to far infrared. Opt. Mater..

[91] Zhao H., Tan Y., Zhang R., Zhao Y., Zhang C.L., Zhang X.C., Zhang L.L. (2021). Molecular dynamic investigation of ethanol-water mixture by terahertz-induced Kerr effect. Opt. Express.

[92] Yuko A., Yasunori T. (2000). Low-frequency Raman study of ethanol-water mixture. Chem. Phys. Lett..

[93] Egashira K., Nishi N. (1998). Low-frequency raman spectroscopy of ethanol-water binary solution: Evidence for self-association of solute and solvent molecules. J. Phys. Chem. B.

